# Effect of Feeding Route and Stroke Type on Gastric Myoelectric Activity in Stroke Survivor Patients: A Preliminary Study

**DOI:** 10.3390/jcm14175976

**Published:** 2025-08-24

**Authors:** Hissah F. Altimyat, Alanoud Aladel, Mahmoud Desoky, Danyah Althuneyyan, Norah Alshammari, Laubna Alagel, Laila Aljabri, Rodan M. Desoky, Mahmoud M. A. Abulmeaty

**Affiliations:** 1 Community Health Sciences Department, College of Applied Medical Sciences, King Saud University, Riyadh 11362, Saudi Arabia; 443204661@student.ksu.edu.sa (H.F.A.); aaladel@ksu.edu.sa (A.A.); 444203384@student.ksu.edu.sa (D.A.); 444203567@student.ksu.edu.sa (N.A.); 444203358@student.ksu.edu.sa (L.A.); 443204548@student.ksu.edu.sa (L.A.);; 2 Internal Medicine, Sultan Bin Abdulaziz Humanitarian City, Riyadh 13571, Saudi Arabia; mdesoky@sbahc.org.sa

**Keywords:** electrogastrography, gastric myoelectric activity, stroke survivors, percutaneous endoscopic gastrostomy, nasogastric tube

## Abstract

**Background/Objectives**: Stroke survivors with dysphagia are usually fed with different feeding routes ranging from oral to percutaneous endoscopic gastrostomy (PEG). However, the impact of the feeding route on the gastric myoelectric activity (GMA) is little-studied. This work examined the effect of feeding route on GMA changes in stroke survivors with dysphagia. **Methods**: This study included 50 patients (20% women) who were divided into three groups based on their feeding route: an oral group (n = 20), a nasogastric group (NGT) (n = 20), and a PEG group (n = 10). For all participants, a nutritional assessment was conducted, and the GMA was measured using a transcutaneous multichannel electrogastrogram (EGG) with a water load satiety test before and after water loading. The EGG-related parameters used in the analysis included the average power distribution by frequency region and the average dominant frequency (ADF). **Results**: The study sample experienced ischemic stroke (66%) or hemorrhagic stroke (34%). At the baseline phase, the PEG group exhibited significantly longer periods of normogastria compared to the NGT and oral groups. Moreover, protein intake was significantly higher in the PEG tube feeding group compared to the other groups. Based on the type of stroke, the ischemic stroke group showed significantly higher tachygastria periods during postprandial EGG recording (*p* = 0.022). The energy and protein consumptions were significantly higher in the hemorrhagic stroke group (*p* = 0.001, *p* = 0.028, respectively). **Conclusions**: The GMA pattern is distinctive for the type of stroke. The PEG feeding route showed more periods with normogastria and the best protein intake.

## 1. Introduction

Every year, 13.7 million strokes occur worldwide, with 60% occurring in individuals under the age of 70 [1], representing a significant burden on public health due to its potential to lead to functional impairment or death [2]. Saudi Arabia has a significant burden of stroke, with the incidence rate expected to double by 2030 [2]. According to the World Health Organization (WHO), stroke is the second leading cause of death in Saudi Arabia [3]. The incidence rate of stroke in Saudi Arabia is 29 strokes per 100,000 individuals annually [4].

A stroke is caused by interruption of the blood supply or rupture of a cerebral blood vessel in a specific region of the brain [5]. Stroke is divided into two main categories, ischemic and hemorrhagic, and it has three stages: the acute stage, which lasts from the time of the stroke’s onset until two weeks later; the subacute stage, which lasts from two weeks onward to six months, and the chronic stage, which lasts longer than six months [6].

Following the stroke, secondary functional alterations in peripheral organs related to the affected cerebral vascular branches should be expected [7,8]. There is substantial clinical and experimental evidence indicating the incidence of certain gastrointestinal (GI) alterations, such as swallowing problems and GI tract dysmotility [9]. Swallowing problems caused by a stroke are known as post-stroke dysphagia (PSD), and it is one of the most feared complications of stroke [10].

Post-stroke dysphagia (PSD) is a leading cause of morbidity, including malnutrition, dehydration, and aspiration pneumonia. It also leads to prolonged hospitalization and increased mortality rate [11,12,13]. Many stroke patients with dysphagia are made to go nothing by mouth (NPO) to restrict oral intake and reduce the risk of aspiration significantly. To meet the dietary and hydration needs of these patients, feeding tubes are commonly inserted [14].

Enteral nutrition is the preferred route, according to both national and international recommendations, and it is initially administered through a nasogastric tube (NGT) for the first 2 to 3 weeks [15]. If nutritional support is required for longer than 4 to 6 weeks, a percutaneous endoscopic gastrostomy (PEG) tube is typically used [15]. Enteral nutrition, beginning with NGT, is the first step for feeding patients with dysphagia, especially in critical care settings, because it is simple, physiological, and affordable [16]. Besides this, enteral feeding with nasogastric tube placement may decrease lower and upper esophageal sphincter function, desensitize the pharyngoglottal adduction reflex, and enhance the frequency of transient lower esophageal sphincter relaxations [17].

There are indications for NGT and PEG feeding, including people post-stroke who fail to take enough fluids and nutrition orally [18]. Stroke survivors who are unable to swallow adequate amounts of food and fluid orally for more than four weeks and are at risk of long-term malnutrition are another indication of PEG feeding [19,20].

Complications that can develop during enteral feeding include mechanical, gastrointestinal, and metabolic issues [21]. The most common complications tend to be related to gastrointestinal function, such as nausea, diarrhea, vomiting, aspiration, high gastric residual volume, abdominal distention, and constipation [21]. Factors such as the feeding route, product used, and feeding frequency may affect the incidence of gastrointestinal complications. Future research is needed to investigate the gastrointestinal complications and related factors in post-stroke dysphagia patients undergoing tube enteral nutrition.

Electrogastrography is a non-invasive technique that utilizes cutaneous electrodes placed on the abdominal skin over the stomach to record gastric myoelectrical activity (GMA), which is the electrical activity that dictates gastric motility [22]. When appropriately recorded, the EGG is a valuable measure of gastric slow waves, providing clinically meaningful information if correctly analyzed and interpreted [22,23]. To date, there have been negligible studies that have used the EGG technique to investigate the connection between alterations in GMA in patients with PSD.

Accumulating clinical and experimental evidence suggests a causal relationship between acute conditions such as stroke and gastric complications [24,25,26]. Thus, it is crucial to determine whether gastric myoelectric dysfunction is triggered by stroke and whether it is associated with other GIT manifestations related to stroke.

The EGG might be a promising method to identify the gastric complications associated with post-stroke dysphagia that develop after ischemic and hemorrhagic types of stroke. Accordingly, this study aimed to capture the changes in GMA in the main types of stroke lesions and investigate the impact of different feeding routes (NGT, PEG, and oral feeding) on GMA changes.

## 2. Materials and Methods

### 2.1. Study Subjects

This cross-sectional study was conducted in the clinical investigation unit (CIU) at Sultan Bin Abdelaziz Humanitarian City (SBAHC), Riyadh, KSA. A total of 50 participants were enrolled. The G* power software (G*Power 3.1.9.7, Heinrich Heine University, Dusseldorf, Germany) was used to calculate the sample size (n of each group = 9) that meets a 95% power, alpha level = 0.05, and an effect size of approximately 2.49, based on the means of GMA arrhythmia percentages in the study by Sun et al. [27]. Recruitment was conducted via pre-booked appointments arranged by the research team. The study included three groups with similar age ranges (35–75 years) of both genders, who had a past history of stroke and were admitted to the hospital during the study period, and were not taking any medications or undergoing surgeries that might affect gastrointestinal function. Patients living with swallowing difficulties that required NGT or PEG feeding, as well as those without swallowing difficulties who were able to tolerate oral feeding, were included. Patients with pharyngeal, esophageal, gastric diseases, or Parkinson’s disease, or with other neurological deficits and incomplete reports were excluded. The participants were divided according to their route of feeding and allocated into three groups: Oral (n = 20), NGT (n = 20), and PEG (n = 10). Informed consent was obtained from every participant after they were informed of the study’s hazards and benefits. One visit was required for every participant. Before the assessment, participants were instructed to fast for at least two hours before the assessment appointment. The work was reviewed and approved by the Institutional Review Board at SBAHC, under reference number 87-2022-IRB.

### 2.2. Demographic Parameters

Demographic and personal data were collected using self-reporting questionnaires, which included age, gender, and marital status. Past medical history of co-morbid conditions was recorded, including cardiovascular disease, diabetes mellitus, hypertension, hyperlipidemia, chronic obstructive pulmonary disease, asthma, previous lung pathology, kidney disease, liver disease, thyroid disease, gastroesophageal reflux disease, peptic ulcer, stroke (stroke type and stroke level), and other comorbid conditions.

### 2.3. Anthropometric Parameters

Body weight was measured by using an electronic scale to the nearest 0.1 kg, and body height was measured using a portable stadiometer to the nearest 0.1 cm. The body mass index (BMI) was calculated as follows: weight (kg)/square of height (m^2^).

### 2.4. Gastrointestinal Manifestation

Manifestations such as vomiting, high gastric residual volume (GRV), diarrhea, and gastrointestinal bleeding were reported. Additionally, dietary parameters such as kcal/day, protein/day, and feeding details, formula type, bolus volume, and frequency were determined.

### 2.5. ANMS GCSI-DD Assessment

The American Neurogastroenterology and Motility Society Gastroparesis Cardinal Symptom Index—Daily Diary (ANMS GCSI-DD), which assesses nausea, early satiety, postprandial fullness, and upper abdominal pain on a severity score from none (0) to very severe (4) and the number of vomiting episodes during the past 24 h, was reported [28].

### 2.6. EGG for Measuring the GMA Activity

An EGG machine integrated with a satiety water-load test was used to measure GMA. The application of the multichannel EEG system was conducted according to previous publications [29,30].

EGG recordings included the baseline (pre-prandial) phase, which commenced once both respiratory and gastric signals had stabilized for at least 10 min, and the postprandial phase, which lasted for 30 min. The two phases were separated by a brief period for the satiety water-load test, during which participants were instructed to sit upright and consume the maximum volume of water tolerable within a 5-minute interval. The volume of ingested water was logged within the EGG software (Version 1.02, 3CPM Co., Sparks Glencoe, MD, USA). The final EGG recording was segmented into four periods: baseline (BL), the first 10 minutes of postprandial recording (Min10), the middle 10 minutes of postprandial recording (Min20), and the last 10 minutes of postprandial recording (Min30).

The EGG device has standardized placement areas for the three electrodes, which are positioned on specific areas of the epigastrium, in addition to a respiratory belt with a sensor to detect movement artifacts [31]. For further minimization of noise and maximization of signal integrity, the device software can detect the good minutes in each recording phase. A minimum of 4 minutes out of 10 minutes is required to give diagnoses.

EGG-derived parameters included ingested water load (WL) volume and spectral power distribution across defined frequency bands within the range of 0–15 cycles per minute (CPM): bradygastria (BradyG) from 1.0 to 2.5 CPM, normogastria (NormoG) from 2.5 to 3.75 CPM, tachygastria (TachyG) from 3.75 to 10 CPM, and duodenal activity (duodenal) 10–15 CPM. Additionally, the average dominant frequency (ADF) was calculated to evaluate the relationship between power spectral density (PSD) and GMA. The ADF was divided into four periods, each period about 10 min (i.e., ADF_10 min, ADF_20 min, ADF_30 min, and ADF_40 min).

### 2.7. Statistical Analysis

The Statistical Package for Social Sciences (SPSS) software, version 25 (IBM, Chicago, IL, USA) was used to analyze these data. The Shapiro–Wilk test was used to test for linearity. Non-categorical data were presented as means ± SD or median (interquartile range) for variables that were not normally distributed. One-way ANOVA with a post hoc LSD test and Bonferroni correction was used to compare continuous variables. In contrast, a non-parametric equivalent (Kruskal–Wallis test) was used to compare the differences in categorical data among the three study groups. Additionally, the independent sample *t*-test or an equivalent non-parametric test was used to compare the ischemic and hemorrhagic stroke groups. A *p*-value less than 0.05 was considered statistically significant.

## 3. Results

### 3.1. Demographic and Descriptive Results

Fifty subjects were included in the study (20% females). The mean age of the participants was 58 (age range ≥ 35 years). The groups showed no significant differences in age, weight, height, BMI, total calorie intake, bolus volume, or time since stroke. The total protein was significantly higher in the PEG group compared to the NGT and oral groups (Table 1).

The stroke type, level, comorbidities, gender, and marital status of the study groups are presented in Table 2. Generally, there were no significant differences between the groups. Moreover, ANMSGCSI-DD parameters and total score are presented in Table 3. Apart from the significantly higher episodes of vomiting in the PEG group, the other parameters and the total score were similar in all study groups.

### 3.2. The EGG Findings Among Subgroups

Table 4 and Table 5 show the EGG recording among the study groups. The baseline assessment revealed statistically significant differences in normogastria among the PEG group compared to the NGT and oral groups. Our findings revealed no significant difference in the period of Min10, Min20, and Min30 parameters (Table 4). Additionally, our findings revealed no significant differences in water load and ADF among the study groups.

The study sample was further divided into two groups based on the type of stroke: the ischemic stroke group (n = 33) and the hemorrhagic stroke group (n = 17). Table 5 presents the GMA patterns according to the type of stroke. The EGG recording at the postprandial Min20 period showed normogastria and tachygastria percentages significantly higher in the ischemic stroke group than in the hemorrhagic stroke group (*p =* 0.049 and 0.015, respectively). However, there was no significant difference in water load volume or ADF.

### 3.3. Anthropometrics and Dietary Intake Findings According to Stroke Type

Table 6 presents anthropometrics and dietary intake according to the stroke type. There was no significant difference in age, Wt, Ht, BMI, or ANMSGCSI-DD Score. Interestingly, the hemorrhagic stroke group had a significantly higher daily calorie consumption (*p =* 0.009) compared to the ischemic stroke group.

## 4. Discussion

Stroke survivors with post-stroke dysphagia (PSD) often complain of gastrointestinal symptoms, leading to poor clinical outcomes and mandating the use of NGT and PEG tube feeding. This study aimed to investigate whether a normal GMA rhythm would change after experiencing different types of stroke and during various feeding routes (NGT, PEG, and oral feeding). This work may provide more scientific information and provide a deep insight into these feeding disabilities.

The mean age of study participants was 58.14 ± 11.18 years, and male participants represented 80% of the sample. It was reported that age, gender, and BMI had no association with the EGG, which was consistent with previous studies [32,33,34,35]. However, our study showed no significant differences in age, weight, height, BMI, total calorie intake, or bolus volume (Table 2).

The findings of this study shed light on the potential use of EGG in the detection of patterns of GMA in post-stroke survivors with dysphagia (PSD) and during feeding them via NGT, PEG, and oral routes. The baseline GMA assessment revealed statistical differences in normogastria times in the PEG group compared with the NGT and oral groups. This was consistent with a previous study conducted before and after PEG tube placement to evaluate the effects of PEG on gastric motility [36]. The study by Ono et al. found that the percentage of normal-range EGGs increased significantly after PEG tube placement [36]. This finding suggests that PEG tube placement may be the optimal feeding route for normalizing gastric myoelectrical activity.

Furthermore, the current study showed a significant difference in the pattern of GMA during the minute-20 period of TachyG, which was high in the ischemic stroke group compared to those with hemorrhagic stroke, while no statistical differences were found in the other GMA patterns. This was consistent with a previous study conducted in post-stroke patients, which found that the most common complication within 2 weeks of the onset of the ischemic stroke was GI dysfunction (39%) [37]

The study’s findings revealed that participants in the hemorrhagic stroke group had a significant increase in daily kilocalorie (Kcal) consumption compared to the ischemic stroke group. Few studies have assessed the association between dietary intake and different types of stroke. A previous study disagreed with this finding, which found no significant difference in Kcal and protein consumption between the hemorrhagic and ischemic stroke groups [38]. However, it is worth noting that they only assessed oral feeding, while our study assessed both tube feeding and oral feeding. The high intake of calories and protein in the hemorrhagic stroke group in this study may not be due to differences in age and body mass index, as these variables were similar in both groups, suggesting that other factors related to the stroke type are at play.

Additionally, the prediction of gastroparesis occurrence was made using ANMS GCSI-DD. The ANMS GCSI-DD was developed as a proactive measure to support a symptom-based endpoint for gastroparesis clinical trials [28]. Previous studies used ANMS GCSI-DD to predict gastroparesis [39,40]. None of these studies combined these variables in the stroke survivor population, as in the current study. The present study found no significant difference in bloating or distension, nausea, post-prandial fullness or early satiety, upper abdominal pain, and ANMSGCSI-DD total score. The only significant result was a significant difference in the frequency of vomiting episodes in the PEG group compared to the NGT and oral groups. However, this result could be influenced by recall bias or be related to the severity of the patient’s condition rather than being directly attributed to gastroparesis.

For the first time, this study measured the GMA and dietary intake among the NGT, oral feeding, and PEG feeding groups in cases with post-stroke dysphagia (PSD) and among different types of stroke. However, the study has certain limitations, including its cross-sectional design and its focus on a single center in Riyadh city. Moreover, stroke severity, severity of dysphagia, other neurological deficits, lesion location/size, and other clinical parameters were not considered for analysis. However, the study participants in all groups were clinically and neurologically similar, as determined by strict inclusion and exclusion criteria.

Despite this, since the sample comprises patients with neurological diseases such as stroke, they often experience poor muscular conditions; some participants may talk, move their hands, or adjust their body posture during the EGG test. This occurs due to the long time needed to complete the research assessment. Notwithstanding, these motion artifacts did not affect our findings because the computerized spectral analysis removed those artifacts, and the physiology consultant reviewed and approved each EGG test or instructed the recording to be repeated.

## 5. Conclusions

This study investigated the value of EGG use in this disease population. The EGG recording at the Min20 period was significantly higher in TachyG percentages in the ischemic stroke group compared to the hemorrhagic stroke group. Furthermore, the PEG group was associated with more normogasteria times. The results can open the door to future research examining gastric motility among stroke patients to improve their feeding choices and dietary intake.

## Figures and Tables

**Table 1:** Anthropometrics and dietary parameters among study groups.

Variables	Oral Group (*n* = 20)	NGT Group (*n* = 20)	PEG Group (*n* = 10)	*p*-Value
Age (years)	54.45 ± 8.81	59.65 ± 13.11	62.50 ± 9.92	0.131
Wt (kg)	73.49 ± 17.11	67.19 ± 30.59	63.33 ± 6.94	0.464
Ht (cm)	167.60 ± 9.22	158.50 ± 25.97	164.80 ± 5.09	0.265
BMI (kg/m^2^)	25.91 ± 4.52	24.05 ± 4.09	23.36 ± 3.09	0.201
Total Kcal (Kcal/day)	1750.0 ± 143.27	1634.75 ± 347.60	1824.0 ± 453.81	0.255
Total Protein (g/day)	76.25 ± 12.16	69.55 ± 17.80	87.70 ± 26.54 *	0.041
Bolus volume (mL/bolus)	0	230.00 ± 33.56	234.0 ± 26.33	0.745
Bolus frequency (bolus/24 h)	0	6.0 ± 00	6.0 ± 00	-
Time since stroke (months)	24.00 (24.00)	24.00 (28.25)	36.00 (15.00)	0.159

BMI: body mass index; cm: centimeter; kg: kilogram; Wt: weight; SD: standard deviation. * significant versus NGT and oral group (*p* = 0.012 by LSD and 0.036 by Bonferroni correction).

**Table 2:** Stroke type and stroke level among study subgroups by using the chi-square test.

Variable	Oral (*n* = 20)		NGT (*n* = 20)		PEG (*n* = 10)		*p*-Value
	%Within Variable	%Within Group	%Within Variable	%Within Subgroup	%Within Variable	%Within Subgroup	
Stroke Type							0.548
Ischemic stroke	36.40%	60.00%	45.50%	75.00%	18.20%	60.00%	
Hemorrhagic stroke	47.10%	40.00%	29.40%	25.00%	23.50%	40.00%	
Stroke level							0.814
Cerebral artery	41.00%	80.00%	38.50%	75.00%	20.50%	80.00%	
Subcortical	40.00%	10.00%	40.00%	10.00%	20.00%	10.00%	
Pontine	33.30%	5.00%	66.70%	10.00%	0.00%	0.00%	
Subarachnoid	50.00%	5.00%	0.00%	0.00%	50.00%	10.00%	
Midbrain	0.00%	0.00%	100.00%	5.00%	0.00%	0.00%	
Comorbidities							0.998
CVD	40.00%	20.00%	40.00%	20.00%	20.00%	20.00%	
DM	40.00%	80.00%	40.00%	80.00%	20.00%	80.00%	
HTN	40.00%	20.00%	40.00%	20.00%	20.00%	20.00%	
Dyslipidemia	40.00%	80.00%	40.00%	80.00%	20.00%	80.00%	
Asthma	40.00%	20.00%	40.00%	20.00%	20.00%	20.00%	
Gender							0.999
Female	40.00%	20.00%	40.00%	20.00%	20.00%	20.00%	
Male	40.00%	80.00%	40.00%	80.00%	20.00%	80.00%	
Marital status							0.417
Single	0.00%	0.00%	50.0%	5.0%	50.0%	10.0%	
Married	40.00%	90.00%	42.00%	95.00%	17.00%	80.00%	
Divorced	67.00%	10.00%	0.00%	0.00%	33.00%	10.00%	

CVD: cardiovascular diseases, DM: diabetes mellitus, HTN: hypertension.

**Table 3:** ANMSGCSI-DD score among study subgroup.

Variables	Oral Group Mean ± SD (*n* = 20)	NGT Group Mean ± SD (*n* = 20)	PEG Group Mean ± SD (*n* = 10)	*p*-Value
Bloating (feeling like you need to loosen your clothes)	1.50 ± 0.88	1.5 ± 0.68	1.4 ± 0.51	0.93
Nausea (feeling sick to your stomach as if you were going to vomit or throw up)	1.5 ± 0.88	1.8 ± 0.58	1.4 ± 0.51	0.17
Not able to finish a normal-sized meal (or bolus)	1.85 ± 0.98	1.45 ± 0.68	1.30 ± 0.48	0.14
Feeling excessively full after meals (or bolus)	1.60 ± 0.88	1.40 ± 0.59	2 ± 1.05	0.18
Upper abdominal pain (above the navel)	1.35 ± 0.67	1.65 ± 0.81	2 ± 1.05	0.12
During the past 24 h, how many episodes of vomiting did you have?	1.10 ± 0.30	1.15 ± 0.48	1.80 ± 0.91 *	0.004
In thinking about your gastroparesis disorder, what was the overall severity of your gastroparesis symptoms today (during the past 24 h)?	1.35 ± 0.81	1.40 ± 0.84	1.40 ± 0.84	0.97
Overall score	3.25 ± 4.01	3.35 ± 2.53	4.30 ± 2.40	0.67

* *p* < 0.05 (2-tailed). SD, standard deviation.

**Table 4:** Electrogastrography (EGG) patterns among oral, NGT, and PEG groups.

Variables	Oral Group Mean ± SD (*n* = 20)	NGT Group Mean ± SD (*n* = 20)	PEG Group Mean ± SD (*n* = 10)	*p*-Value
WL volume	236.50 ± 88.87	215.0 ± 89.05	183.0 ± 66.51	0.275
BL_BradyG	53.72 ± 31.98	51.69 ± 32.53	48.58 ± 28.58	0.915
BL_NormoG	9.49 ± 5.85	7.71 ± 6.45	19.95 ± 25.48 *	0.041
BL_TachyG	24.37 ± 20.05	29.75 ± 21.27	25.76 ± 15.72	0.682
BL_Duodenal	12.47 ± 12.90	10.42 ± 11.96	11.11 ± 10.20	0.863
Min10_BradyG	62.09 ± 28.08	61.43 ± 33.82	61.30 ± 30.61	0.997
Min10_NormoG	8.40 ± 6.78	7.86 ± 5.46	12.63 ± 17.47	0.403
Min10_TachyG	21.22 ± 18.52	24.32 ± 23.19	17.74 ± 15.51	0.692
Min10_Duodenal	8.33 ± 9.70	7.63 ± 10.05	8.33 ± 10.71	0.971
Min20_BradyG	51.17 ± 31.85	50.59 ± 34.32	57.74 ± 33.93	0.843
Min20_NormoG	10.55 ± 7.95	9.40 ± 6.51	14.78 ± 17.63	0.387
Min20_TachyG	28.10 ± 21.73	25.82 ± 19.54	22.82 ± 22.01	0.806
Min20_Duodenal	10.17 ± 11.87	11.03 ± 13.83	4.66 ± 6.42	0.369
Min30_BradyG	49.84 ± 26.37	42.84 ± 28.50	61.39 ± 36.64	0.275
Min30_NormoG	8.96 ± 4.95	8.54 ± 6.19	8.100 ± 13.15	0.981
Min30_TachyG	30.59 ± 17.70	35.05 ± 16.59	20.48 ± 22.75 **	0.133
Min30_Duodenal	11.35 ± 8.72	13.58 ± 12.67	9.14 ± 13.66	0.595
ADF_10 min	3.62 ± 4.40	2.54 ± 2.78	1.52 ± 0.70	0.256
ADF_20 min	6.07 ± 16.47	1.81 ± 1.47	1.47 ± 0.84	0.361
ADF_30 min	5.22 ± 11.04	2.83 ± 2.35	2.12 ± 1.72	0.445
ADF_40 min	2.49 ± 4.40	1.25 ± 2.16	2.34 ± 2.92	0.472

WL, water load; BL, baseline; BradyG, bradygastria; NormoG, normogastria; TachyG, tachygastria; ADF: average dominant frequency. * indicates that the baseline normogastria percentage in the PEG group was significantly higher than that in the Oral and NGT groups (*p =* 0.035 and 0.015, respectively, by LSD) and significantly higher than the NGT group only (*p =* 0.044 by Bonferroni correction). ** indicates that the Min30 tachygastria percentage in the PEG group was significantly lower than that in the NGT groups (*p =* 0.046 by LSD) and insignificantly changed (*p =* 138 by Bonferroni correction).

**Table 5:** Gastric myoelectrical activity parameters and stroke type.

Variable	Ischemic Stroke Mean ± SD (*n* = 33)	Hemorrhagic Stroke Mean ± SD (*n* = 17)	*p*-Value *
WL Volume	221.82 ± 82.74	208.24 ± 93.09	0.601
BL_BradyG	53.10 ± 30.11	49.52 ± 33.46	0.710
BL_NormoGB	9.49 ± 11.08	13.55 ± 16.26	0.401
BL_TachyG	25.44 ± 18.88	29.44 ± 21.19	0.519
BL_Duodenal	11.75 ± 12.97	10.66 ± 9.57	0.733
Min10_BradyG	58.10 ± 33.04	68.60 ± 23.69	0.199
Min10_NormoG	9.31 ± 10.64	8.48 ± 6.72	0.730
Min10_TachyG	23.83 ± 21.98	17.76 ± 14.42	0.237
Min10_Duodenal	9.51 ± 11.19	5.22 ± 5.75	0.098
Min20_BradyG	46.59 ± 31.24	63.25 ± 33.59	0.085
Min20_NormoG	12.83 ± 11.26	7.26 ± 6.11 ^#^	0.049
Min20_TachyG	30.87 ± 21.30	16.94 ± 15.99^#^	0.015
Min20_Duodenal	9.70 ± 11.50	8.84 ± 13.07	0.822
Min30_BradyG	47.29 ± 29.01	53.35 ± 31.32	0.500
Min30_NormoG	9.73 ± 8.04	6.100 ± 6.08	0.198
Min30_TachyG	30.44 ± 18.23	30.19 ± 20.35	0.962
Min30_Duodenal	13.06 ± 12.02	9.37 ± 9.80	0.251
ADF_10 min	2.94 ± 3.77	2.43 ± 2.39	0.558
ADF_20 min	2.17 ± 2.41	5.91 ± 17.84	0.474
ADF_30 min	4.23 ± 8.53	2.52 ± 3.28	0.414
ADF_40 min	2.21 ± 4.02	1.49 ± 1.31	0.371

BL, baseline; BradyG, bradygastria; NormoG, normogastria; TachyG, tachygastria; ADF: average dominant frequency. * *p*-value after Bootstrapping for 1000 samples. ^#^ Statistically significant.

**Table 6:** Anthropometrics and dietary parameters among stroke type groups.

Variable	Ischemic Stroke Mean ± SD (*n* = 33)	Hemorrhagic Stroke Mean ± SD (*n* = 17)	*p*-value *
Age (years)	58.91 ± 10.95	56.65 ± 11.84	0.504
Wt (kg)	70.20 ± 25.00	66.48 ± 16.61	0.552
Ht (cm)	161.94 ± 20.84	166.24 ± 9.39	0.387
BMI (kg/m^2^)	25.11 ± 4.38	23.77 ± 3.65	0.248
ANMSGCSI-DD Score	3.48 ± 3.22	3.53 ± 3.12	0.963
Total Kcal (kcal/day)	1618.18 ± 216.70	1913.82 ± 381.38 ^#^	0.009
Total Protein (g/day)	71.70 ± 14.98	83.4 ± 23.06	0.064

BMI: body mass index; cm: centimeter; kg: kilogram; Wt: weight; SD: standard deviation. * *p*-value after bootstrapping for 1000 samples. ^#^ Statistically significant.

## Data Availability

Original data supporting these results are available on request from the corresponding author for reasonable purposes.
